# Perceptions of general practitioners towards the services provided by advanced practice nurses: a cross-sectional survey in France

**DOI:** 10.1186/s12913-023-10420-y

**Published:** 2023-12-20

**Authors:** Charles Goddaert, Pierre-Antoine Gérard, Charlotte Kessler, Mélaine Leblanc, Coralie Barbe, Jan Chrusciel, Clément Cormi, Stéphane Sanchez

**Affiliations:** 1 Department of General Medicine, Faculty of Medicine, University of Reims Champagne Ardennes, Marne, France; 2 Department of Advanced Practice, University of Reims Champagne Ardennes, Reims, Marne, France; 3 Public Health and Performance Department, Champagne Sud Hospital, Troyes, Aube, France; 4 University Committee of Resources for Research in Health (CURRS), University of Reims, Marne, France

**Keywords:** Advanced practice nurse, General practitioner, Perceptions, Access to care, Primary care

## Abstract

**Background:**

New healthcare professions are emerging due to scarce medical resources. The appearance of a new healthcare profession, advanced practice nurses (APNs), has raised questions about how general practitioners interrelate with them as primary care providers. The objective of this study was to explore the perceptions general practitioners have towards the services rendered by APNs to patients, to general practice and the role they play in the healthcare system.

**Methods:**

A survey-based, cross-sectional study was conducted throughout the *Grand Est* region of France which covers 57,333km^2^ and has a population of approximately 5,562,651. The survey was compiled using pre-existing questionnaires and was carried out from July to September 2022 via email. Variables collected were rate of acceptability and socio-demographic characteristics.

**Results:**

In total, 251 responses were included. The mean age of general practitioners was 41.7 years, most were women (58.2%) and worked in rural areas of the region (53.8%). Over 80% of respondents practiced in group structures (defined as either multi-professional health centers (n = 61) or in group practices (n = 143)). Most respondents (94.0%) were familiar with the APN profession and did not consider that APNs improved access to care (55.8%, percent of responders with score ≤ 3/10). Moreover, most did not believe that APNs were useful as a primary care provider for patients (61.8%). However, being a member of a territorialized healthcare community, known as *Communautés Professionnelles Territoriales de Santé* (CPTS*)*, was associated with a positive appraisal of APNs’ services (OR = 2.116, 95%CI: 1.223 to 3.712; *p* = 0.007).

**Conclusions:**

Encouraging shared and networked practice within a healthcare community may promote a positive perception of new actors. Further studies need to be conducted to show whether the integration of APNs into healthcare networks improves quality of care.

**Supplementary Information:**

The online version contains supplementary material available at 10.1186/s12913-023-10420-y.

## Background

Population aging has increased the prevalence of chronic diseases leading to a rise in the demand for healthcare services and strain on healthcare systems [1, 2]. By 2040, the supply of general practitioners (GPs) in France is anticipated to decline despite the growing number of responsibilities assigned to them [3, 4]. Moreover, French GPs are increasingly reluctant to take on new patients. A national longitudinal survey pointed out that 65% of GPs refused new patients in 2022 compared to 53% in 2019 [5]. Similar observations have been made in other countries including Ireland where care has been increasingly moved from a hospital setting to the city despite a GP shortage [6]. Most evidently, a lack of GPs is found in elderly care in which the use of new strategies have been required to create more “time for care” [7].

To date, these strategies have encompassed measures including the use of telemedicine [8] to reduce unnecessary consultations and ease the administrative burdens placed on physicians [9]. Another solution has been to substitute GPs with paramedical personnel or advanced practice nurses (APNs) for specific indications. APNs are nurses prepared at a master’s level for advanced clinical practice. In France, they are authorized for first-time prescriptions within a defined scope (stable chronic conditions in primary care, oncology, psychiatry, and emergencies). In comparison, other nursing roles are only given the role of providing refills [10].

According to research, this substitution has demonstrated comparable or even superior outcomes [11, 12], depending on the responsibilities taken by APNs (including managing hypertension), while reducing costs for care [13, 14]. However, effective implementation of such a substitution depends on the collaborative relationship between physicians and APNs in terms of trust, communication, proactive engagement and more notably, the recognition of APNs as autonomous healthcare professionals (HCPs) [15]. In the United Kingdom (UK), nurse-led care is well established, with nurses progressively taking on increasingly advanced roles such as having the responsibility of providing prescriptions which was previously met with occasional anxiety or concern by physicians [16–18].

In France, the legal and educational framework for APNs was established in 2016 with the first tertiary qualifications granted in 2019 [19, 20]. Even if French citizens opined that the role of APNs is positive [21], questions have been raised regarding its integration and reception within the French GP community and overall role in general practice. Some have viewed the APN profession as a response to shortcomings in previous healthcare policies, whereas others have expressed the potential risks involved in outsourcing medical services to independent contractors. There are also concerns that a shift in general practice may add unnecessary complexity [22, 23] with a polarized debate in the media surrounding the adoption of APNs and the experiences of other countries that have integrated APNs into healthcare systems over the years [24]. Lastly, APNs are sometimes seen as competitors of GPs who fear seeing their activity decline. The objective of this study was to describe the perceptions GPs had of the services rendered by APNs in terms of their impact on GPs themselves, on patients, on the overall healthcare system as well as additional factors associated with their perceptions.

## Methods

### Study design

A cross-sectional study based on a 20-item questionnaire was conducted from July to September 2022. The questionnaire was distributed online using the Lime Survey platform (https://www.limesurvey.org). To ensure content validity, the questionnaire was developed after a comprehensive review of literature related to APN practices by a panel of three experts consisting of GPs and public health physicians.

The first eight items of the questionnaire focused on the sociodemographic characteristics of the respondents, including their practice setting (urban or rural) and whether they practiced individually or in a group setting. The remaining items (Questions 9 to 20) assessed GPs perspective of the services provided by APNs, namely their impact on general practice (Questions 9 to 11), on patients (Questions 12 to 14) and the organization of care as well as on the impact they have on the healthcare system (Questions 15 to 20). Most items were assessed using a rating scale ranging from 1 (Not useful at all) to 10 (Very useful), or required binary responses (Yes/No). The complete questionnaire can be found in the Supplementary Material.

All GPs who were members of the *Union Régionale des Professionnels de Santé de la région Grand-Est (URPS Grand-Est)* were initially contacted via email to participate in the study. The *URPS Grand-Est* is a professional union that represents the interests of private practicing HCPs in the Grand-Est region and participates in the organization of healthcare within the region. Three reminder emails were subsequently sent at 15-day intervals. This study adhered to the Checklist of Reporting Results of Internet-E-Survey (CHERRIES) guidelines to ensure methodological rigor and transparency in reporting the findings [25].

### Inclusion/Exclusion criteria

All private practice GPs in the region who were members of the *URPS Grand-Est* and provided consent to participant in the study were included. Those who refused consent were excluded from the study.

### Statistical analysis

Continuous variables were described as mean ± standard deviation (SD), while categorical variables were expressed as numbers and percentages (%). The perception of the services rendered by APN to general practice was defined as the sum of the scores of three questions that were considered by the expert review to measure a similar construct regarding services rendered by APNs to GPs. These questions were: (i) perceived usefulness of working with an APN as a team (maximum 10 points), (ii) perceived usefulness of the APN to decrease the physicians’ workload (maximum 10 points), and (iii) perceived usefulness of the APN for the follow-up of patients with chronic diseases (maximum 10 points). The sum of question scores was divided by three so that the maximum score for this construct was 10. A correlation coefficient between the results of each of the individual underlying questions (measured by Spearman’s correlation coefficient) was estimated to confirm that each of the underlying questions measured a related concept.

To make the analysis easier to interpret, the summary measure of the perception of the services rendered by APNs to general practice was then dichotomized at the median (in this particular case, dichotomization at the mean would have provided identical results because the variable was symmetrically distributed around the mean). Scores below the median were categorized as “low perception of services rendered by APNs”, whereas scores equal to or above the median were classified as “positive perception of services rendered by APNs”. Chi-squared tests and Student’s *t* tests were performed to compare the characteristics of groups based on their perceived satisfaction with the services rendered by APNs. Categorial variables were reported with the unadjusted odds ratios (OR) along with the corresponding 95% confidence interval (CI).

A multiple logistic regression model was fitted, estimating the probability of a positive perception of services rendered by APN to general practice. The variables entered in the model were chosen based on an expert review of clinical relevance. The multivariate analysis was performed with the following variables: age, sex, place of practice and whether the GP was a member of a territorialized healthcare community, known as *les Communautés Professionnelles Territoriales de Santé* (CPTS). Model performance was evaluated using the Area Under the Curve (AUC).

A second construct, termed “positive perception of services rendered to patients in primary care”, was obtained by summing the responses of the questions: “How useful do you perceive APNs are regarding the improvement of patient access to healthcare?” and “How useful do you perceive APNs are as a first resort for stabilized patients?”. The result was divided by two in order for the construct to have a theoretical maximum of 10 points. A Spearman correlation coefficient was performed on these variables to confirm that they were measuring a similar underlying concept. As this construct was a secondary outcome, the variables associated with this summary score were solely explored in a univariate analysis. A median split was also performed on this variable so that the results could be conveyed as a comparison between distinct groups according to the perceptions of APNs usefulness. Statistical analyses were performed using R version 4.2.3 (R Foundation for Statistical Computing, Vienna, Austria). A p-value less than 0.05 was considered statistically significant.

## Results

A total of 251 out of 4,357 GPs participated in this study (response rate: 5.8%). Among the respondents, the mean age was 41.7 years and 58.2% were women. In terms of practice location, 53.7% (n = 135) reported their general practice was located in a rural area. More than 80% of the participants (n = 204) practiced in group healthcare structures including multi-professional health centers (n = 61) and group practices (n = 143). Most respondents (94.0%) indicated familiarity with the APN profession.

### Service rendered by APNs to general practice

Among the respondents, half of the GPs expressed the belief that working as a team with an APN was not helpful (the median score for this question was 5 out of 10 possible points, Q1 to Q3, 2 to 8). Similarly, responders appeared to have mixed opinions regarding the usefulness of APNs for the long-term follow-up of patients with stable chronic diseases (median score: 5, Q1 to Q3, 2 to 8). Most GPs did not believe that APNs would reduce their workload, as expressed by low scores on the corresponding question: median 3 out of 10 (Q1 to Q3 1 to 6). Pairwise Spearman correlation coefficients between the three above-mentioned variables were all superior to 0.80, confirming that these variables measured a related underlying concept.

The main outcome of a favorable perception of the service provided by APNs for general practice, defined as a score superior or equal to the median score for the sum of the three corresponding questions (normalized to a maximum value of 10), was defined using a cut-off value of 4.7.

In the univariate analysis, the grouped type of practice (vs. the single type) was significantly associated to a more favorable perception of the service provided by APNs for general practice (Odds Ratio OR = 1.923 [95% CI: 1.004 to 3.681]) (Table 1). GPs who were CPTS members (territorialized healthcare community) also exhibited a significantly more positive perception of the service rendered by APNs for general practice (OR = 2.005 [95% CI: 1.179–3.407]).

**Table 1:** Perceptions of general practitioner’s (GPs) on the services rendered by advanced practice nurses (APNs) in general practice (univariate analysis)

GP Characteristics	Total, n (%)	Perception of GPs towards the services rendered by APNs, n (%)		Unadjusted OR (95% CI)	*p*-value
		**Low perception** **(< Median value of 4.7)**	**Positive perception** **(≥ Median value of 4.7)**		
Sex				0.771 (0.466 to 1.275)	0.31
*Male*	105 (41.8)	55 (52.4)	50 (47.6)		
*Female*	146 (58.2)	67 (45.9)	79 (54.1)		
Age (years)		42.8 (10.8)	40.7 (9.4)		**0.09**
Years in practice		10.1 (8.9)	9 (8.1)		0.33
Place of practice				0.793 (0.482 to 1.303)	0.36
*Urban*	116 (46.2)	60 (51.7)	56 (48.3)		
*Rural*	135 (53.8)	62 (45.9)	73 (54.1)		
Type of practice				1.923 (1.004 to 3.681)	**0.046**
*Group practice*	204 (81.3)	93 (45.6)	111 (54.4)		
*Private (single practice)*	47 (18.7)	29 (61.7)	18 (38.3)		
Professional and territorial health community* membership				2.005 (1.179 to 3.407)	**0.01**
*Yes*	88 (35.1)	33 (37.5)	55 (62.5)		
*No*	163 (64.9)	89 (54.6)	74 (45.4)		
Knowledge of APNs				0.921 (0.323 to 2.620)	**0.88**
*Yes*	236 (94.0)	115 (48.7)	121 (51.3)		
*No*	15 (6.0)	7 (46.7)	8 (53.3)		

APN; Advanced Practice Nurses, *in the original language: *Communautés Professionnelles Territoriales de Santé (CPTS)*

In the multivariate analysis, we found a significant association between a GP being a CPTS member and having a more positive perception of APNs. Specifically, GPs belonging to a CPTS member were approximately twice as likely to have favorable views towards the services rendered by APNs than GPs who did not belong to a territorial healthcare community (OR = 2.116 [95% CI: 1.223 to 3.712]; *p* = 0.007) (Table 2).

**Table 2:** Perceptions of general practitioner’s (GPs) on the services rendered by advanced practice nurses (APNs) in the field of general practice (multivariate analysis)

GP characteristics	OR (95% CI)	*p*-value
Age (numeric variable; OR for each additional year)	0.976 (0.951 to 1.000)	0.054
Sex (male, reference: female)	0.702 (0.416 to 1.179)	0.181
Place of practice (urban, reference: rural)	0.859 (0.51 to 1.449)	0.569
Professional and territorial health community membership	2.116 (1.223 to 3.712)	0.007

OR: Odds Ratio, AUC: 0.629

### Services rendered by APNs to patients

Most physicians responded with relatively low scores when asked if APNs would be useful in improving access to care: median 3 (for a maximum of 10 points; Q1 to Q3, 1 to 6). Numerous respondents did not believe that APNs were beneficial as the initial point of contact for patients with stable chronic diseases: the median score for this question was only 2 (for a maximum of 10; Q1 to Q3, 1 to 5). Answers provided for these two questions were highly correlated (Spearman correlation coefficient 0.796), which was in favor of the hypothesis that they both measured a related construct. Overall, the median of the resulting summary score regarding the perception of services provided by APNs to patients was relatively low, indicating that responders were not convinced by the potential offered by APNs for the early treatment of their patients (median 3 out of 10 possible points, Q1 to Q3, 1 to 5.75).

GPs who practiced alone tended to express a less favorable perception of the service provided by APNs to patients than those who practiced in a group setting. Specifically, 59.6% of GPs practicing alone expressed a poor perception of APN services, compared to 45.1% of those practicing in a group setting. However, this difference did not reach statistical significance (*p* = 0.08) (Table 3).

**Table 3:** Perceptions of general practitioner’s (GPs) on the services rendered by advanced practice nurses (APNs) towards patients (univariate analysis)

GP Characteristics	Total (n = 251) n (%)	Perception of GPs towards the services rendered by APNs towards patients		Unadjusted OR (95% CI)	*p*-value
		**Low perception** **(< median value of 3)**	**Higher perception** **(≥ median value of 3)**		
Sex				0.949 (0.574 to 1.568)	**0.84**
*Male*	105 (41.8)	51 (48.6)	54 (51.4)		
*Female*	146 (58.2)	69 (47.3)	77 (52.7)		
Mean age (years)		42.50 (10.1)	40.98 (10.2)		**0.24**
Years in practice		9.77 (8.3)	9.29 (8.7)		**0.66**
Place of practice				0.906 (0.551 to 1.489)	**0.70**
*Urban*	116 (46.2)	57 (49.1)	59 (50.9)		
*Rural*	135 (53.8)	63 (46.7)	72 (53.3)		
Type of practice				1.794 (0.942 to 3.418)	**0.08**
*Group practice*	204 (81.3)	92 (45.1)	112 (54.9)		
*Private (single practice)*	47 (18.7)	28 (59.6)	19 (40.4)		
Professional and territorial health community* membership				1.241 (0.737 to 2.089)	**0.42**
*Yes*	88 (35.1)	39 (44.3)	49 (55.7)		
*No*	163 (64.9)	81 (49.7)	82 (50.3)		
Knowledge of APNs				0.070 (0.009 to 0.543)	**0.01**
*Yes*	236 (94.0)	119 (50.4)	117 (49.6)		
*No*	15 (6.0)	1 (6.7)	14 (93.3)		

APN; Advanced Practice Nurses, *in the original language: *Communautés Professionnelles Territoriales de Santé (CPTS)*

### Services of APNs rendered to the healthcare system

Most respondents did not think that the APN profession was innovative or that it would contribute to the enhancement of patient care within existing healthcare systems (median score 3 out of 10, Q1 to Q3, 1 to 6). Moreover, responding physicians did not consider that the APN profession would be useful compared to other nursing professions or medical assistants, with a median rating of two out of ten (Q1 to Q3, 1 to 5).

GPs also reported low confidence regarding the probability that APNs would improve communication and collaboration between HCPs: median 2 (out of 10 possible points; Q1 - Q3 1–5). As much as 59.8% of the respondents (150/251) held the opinion that patient care pathways were already crowded as is, with numerous and varying HCPs. Moreover, most responders did not agree that the APN would contribute to a reduction of healthcare costs and expenditure (210 negative answers/251 responders: 83.7%).

## Discussion

In this study, we found that GPs perceived the services provided by APNs as not useful or minimally useful to general practice. In 2021, Aghnatios et al. conducted a comparative analysis of stakeholder perceptions towards the APN profession among patients, GPs, self-employed nurses and APN students [26] and found that GPs expressed the greatest hesitation, stating that there had been no consultation on the role to be played by APNs. In a systematic review, Jakimowicz et al. (2017) emphasized the necessity of establishing and maintaining a trusting relationship between GPs and APNs [27]. Given the limited understanding of APNs’ responsibilities by GPs, establishing productive collaborations may require considerable time. However, this could be facilitated through collaborative initiatives [27–29]. Considering that the first graduation of APNs in France was in 2019, a potential shift in the perceptions of GPs towards APNs could occur in the years to come.

Current care models are facing challenges in terms of sustainability primarily due to factors such as population aging, rise in chronic diseases as well as the occurrences of epidemics and pandemics [24]. Given the strain on medical resources, physicians are increasingly assuming a supervisory role over other HCPs especially in primary care [24]. Since primary care is patient-centric (rather than disease centered), first-contact care could be provided by trained paramedical personnel or APNs [30]. Therefore, contrary to the perceptions reported in our study, certain tasks and responsibilities such as screening and preventive activities could be appropriately delegated to APNs and do not entail competition in general practice [31, 32].

Our results showed that rural GPs consider the services rendered by APNs to be more significant, when compared to their urban counterparts. As shown in literature, prioritizing the deployment of APNs in rural regions may be a suitable approach to familiarize patients with the profession in France [33–35].

According to our survey, the integration of APNs was not perceived by GPs as a good method to enhance access to care. However, this approach is considered to have merit by other actors: a £15 million national investment in the United Kingdom (UK) was certainly based on the assumption that APNs would shorten appointment wait times and provide physicians with more dedicated medical time [9]. It may be worth noting that the system of integrating APNs does not necessarily result in reduced working hours for GPs since it requires supervision and additional organization and meetings [27, 36]. Therefore, a multi-professional teamwork setting could be encouraged [37]. One such initiative aimed to foster inter-professional cooperation within the same geographical area is the establishment of territorial healthcare communities, and our study reflected this since the GPs actively participating in these communities were more likely to perceive APNs as beneficial for both themselves and their patients. This is in line with the results of a study conducted in New Zealand, that shows that managers of general practices working with nurse practitioners (which could be more likely in broader healthcare communities) had a more positive perceptions of their role [38].

Regarding the management of chronic diseases, the literature suggests that compared to GPs, APNs can demonstrate better outcomes in monitoring arterial hypertension [39] and similar outcomes for HbA1c levels in diabetic patients [11, 40]. Similarly, APNs were found to be more effective in preventive care since they could allocate more time to the tasks involved because of the narrower scope of their responsibilities in comparison to GPs [27, 31].

The participants in our study expressed difficulty in understanding the role of APNs within the existing healthcare service landscape. It is worth noting that satisfactory task delegation protocols already exist between physicians and nurses. This may have led respondents to perceive the APN profession as a duplication of existing work structures (3). This perception may likely influence their assessment of the cost/benefit ratio associated with this profession. However, there is currently no evidence in literature suggesting that the integration of APNs marks an inflationary effect on healthcare system costs [11, 40].

### Limitations

The main limitation of our study pertained to the characteristics of our sample. Participants that responded to the survey were predominantly comprised of female GPs and with a younger age that does not correspond with the mean demographics of GPs both in France and in the *Grand-Est* region specifically. Moreover, the low response rate may have imposed a selection bias that may have led to an overrepresentation of GPs who exhibited reluctance towards the integration of APNs in general practice. Considering the nascent nature of the APN profession in France, it is anticipated that the perception of APNs by GPs could evolve when they become more accustomed to the presence of APNs in healthcare practices.

## Conclusions

The primary care system in France is currently undergoing significant transformations and the integration of APNs holds promise. GPs who answered the survey in this study had an overall unfavorable perception of the APN profession, except for those who were accustomed to collaborating within multidisciplinary teams. Although other studies have shown that their point of view is not shared by other physicians, our findings show the persistence of an active subgroup of physicians who do not consider APNs as helpful or useful to patients or to the healthcare system. These results may allow GPs to have a better understanding about the advantages of collaborating with APNs. Further studies need to be conducted to show whether the integration of APNs into healthcare networks improves quality of care which could also be communicated to all stakeholders, especially GPs, in order to ensure a greater cooperation between traditional and newer healthcare settings.

### Electronic supplementary material

Below is the link to the electronic supplementary material.

Supplementary Material 1

## Data Availability

The data analyzed in this study is subject to the following licenses/restrictions: these data were provided especially for the purposes of this study. They are therefore not available to the public. Requests to access these datasets should be directed to SS via stephane.sanchez@hcs-sante.fr.

## List of abbreviations

APN: advanced practice nurse
AUC: Area Under the Curve
CPTS: *Communautés Professionnelles Territoriales de Santé*
HCP: Healthcare professional
GP: General practitioner
