# Supplementary material for: Perceptions of general practitioners towards the services provided by advanced practice nurses: a cross-sectional survey in France
Source: BMC Health Serv Res. 2023 Dec 20;23:1442. doi: 10.1186/s12913-023-10420-y (PMC10734111; doi:10.1186/s12913-023-10420-y)
Supplement: Supplementary file 1 — Supplementary Material 1 [file 12913_2023_10420_MOESM1_ESM.docx]

**Supplementary Material**

| **Questionnaire**  Hello, my name is Pierre-Antoine Gérard and I am a medical student specializing in general practice at the Faculty of Medicine, University of Reims Champagne Ardennes in France. The subject of my thesis is the perception of general practitioner on the services rendered by advanced practice nurses (APNs) in the Grand Est Region of France.  APNs are registered nurses who have acquired theoretical knowledge, as well as the practical know-how to make complex decisions in addition to the clinical skills essential to their profession. The characteristics of APNs are determined by the context in which these nurses are authorized to practice. In France, APNs are authorized to monitor patients with stable chronic pathologies.  There has been much debate among general practitioners concerning the establishment of this new profession at the heart of the healthcare system. This questionnaire will part of a study aimed to describe your perceptions on this topic.  Please answer the following 20 questions separated into two sections (estimated time: 3 to 5mins).  **General Information**   1. What is your gender? Male/Female/Other 2. What is your age? 3. What is your postal code? 4. Location of practice? Rural/urban 5. What is your mode of general practice? Interprofessional healthcare center/group practice/independent 6. Do you have a membership in a local professional healthcare community (CPTS)? Yes/No 7. How many years have you been practicing at this facility? 8. Are you familiar with the advanced practice nursing profession? Yes/No   **Perceptions of APNs**  ***Questions regarding the integration of APNs to general practice***   1. On a scale of 1 to 10 with 1 being ‘not useful at all’ and 10 being ‘extremely useful’, how useful do you perceive it is to work with a team that includes an APN? 2. On a scale of 1 to 10 with 1 being ‘not useful at all’ and 10 being ‘extremely useful’, how useful do you perceive APNs are in reducing the workload in general practice? 3. On a scale of 1 to 10 with 1 being ‘not useful at all’ and 10 being ‘extremely useful’, how useful do you perceive APNs are in monitoring stable, long term, chronic patients?   ***Questions regarding the services rendered by APNs to patients***   1. On a scale of 1 to 10 with 1 being ‘not useful at all’ and 10 being ‘extremely useful’, how useful do you perceive APNs are regarding the improvement of patient access to healthcare? 2. On a scale of 1 to 10 with 1 being ‘not useful at all’ and 10 being ‘extremely useful’, how useful do you perceive APNs are as a first resort for stabilized patients? 3. If you were to work with an APN, would you be concerned about losing your social connection to your long-term patients? Yes/No   ***Questions regarding the impact of the services provided by APNs to the healthcare system***   1. On a scale of 1 to 10 with 1 being ‘not at all likely’ and 10 being ‘extremely likely’, how do you perceive APNs are as innovative professionals that will improve patient care within the current healthcare system? 2. On a scale of 1 to 10 with 1 being ‘not at all likely’ and 10 being ‘extremely likely’, how do you perceive APNs as being able to facilitate exchanges between healthcare professionals (the connection between the city and hospital for example)? 3. On a scale of 1 to 10 with 1 being ‘not at all likely’ and 10 being ‘extremely likely’, how do you perceive APNs as being useful in the current healthcare system compared to teams comprised of private practice ASALEE nurses and medical assistants within general practice? 4. On a scale of 1 to 10 with 1 being ‘no threat at all’ and 10 being ‘an extreme threat’, how do you feel that the APN profession is a threat to your profession and to general practice? 5. Do you think that including APNs into general practice might be involving too many healthcare professionals in the patient care pathway? Yes/No 6. Do you think that APNs reduce healthcare costs? Yes/No   Thank you for your participation. |
| --- |
